# Income, personality, and subjective financial well-being: the role of gender in their genetic and environmental relationships

**DOI:** 10.3389/fpsyg.2015.01493

**Published:** 2015-09-29

**Authors:** Michael J. Zyphur, Wen-Dong Li, Zhen Zhang, Richard D. Arvey, Adam P. Barsky

**Affiliations:** ^1^Department of Management and Marketing, The University of MelbourneMelbourne, VIC, Australia; ^2^Department of Psychological Sciences, Kansas State University, Manhattan, KSUSA; ^3^Department of Management, Arizona State University, Tempe, AZUSA; ^4^Department of Management and Organization, National University of SingaporeSingapore, Singapore

**Keywords:** subjective financial well-being, gender, income, core self evaluations, structural equation modelling, survey of midlife development in the U. S.

## Abstract

Increasing levels of financial inequality prompt questions about the relationship between income and well-being. Using a twins sample from the Survey of Midlife Development in the U. S. and controlling for personality as core self-evaluations (CSE), we found that men, but not women, had higher subjective financial well-being (SFWB) when they had higher incomes. This relationship was due to ‘unshared environmental’ factors rather than genes, suggesting that the effect of income on SFWB is driven by unique experiences among men. Further, for women and men, we found that CSE influenced income and SFWB, and that both genetic and environmental factors explained this relationship. Given the relatively small and male-specific relationship between income and SFWB, and the determination of both income and SFWB by personality, we propose that policy makers focus on malleable factors beyond merely income in order to increase SFWB, including financial education and building self-regulatory capacity.

## Introduction

Income inequality is increasing at an alarming rate and may even be a necessary outcome of capitalism (Picketty, 2014). At the individual level, the question of income inequality is partly about income’s effect on well-being. As this effect increases, income inequality creates well-being inequality, which has implications for social welfare and fairness in society.

To better understand this effect, we study a facet of subjective well-being (SWB) that is important for SWB and directly relevant to income (Ng and Diener, 2014): subjective financial well-being (SFWB). Although different fields treat SFWB differently (e.g., Kushman and Ranney, 1990; van Praag and Frijters, 1999; Joo and Grable, 2004; Penn, 2009), from a psychological perspective SFWB can be defined as a general attitude about one’s financial situation, including overall satisfaction with it but also perceived financial strains, perceived manageability of finances, and perceived financial prospects. SFWB is important because it is linked to income and financial decision-making (van Praag et al., 2003), including job choices and career outcomes (e.g., Judge et al., 2010).

Although research into SFWB is sparser than that on SWB, literature on the income-SWB relationship shows similar findings and describes SWB’s stable antecedents such as personality (DeNeve and Cooper, 1998; Diener and Lucas, 1999) and the social comparison processes that keep the SWB-income relationship small in developed nations (Clark and Oswald, 1996). However, only a few studies integrate these foci by studying whether genetic and environmental influences explain the *relationships* among personality, income, and SWB (e.g., Johnson and Krueger, 2006). We empirically study this question and contribute to the extant literature by focusing on gender differences, investigating the genetic and environmental relationships among personality, income, and SFWB for men and women.

We motivate our study by noting that past research shows a different relationship between income and SWB for men versus women, and this difference may be related to “psychological factors such as needs, desires, and role” (Crowley, 1998; Diener and Biswas-Diener, 2002, p. 131). In turn, because “needs, desires, and role” are a function of genetic and environmental factors, there remains a question regarding how a gender difference in the income-SWB relationship relates to genetic and environmental factors.

To investigate this question, in what follows we first describe existing theory and findings for SWB, and then focus more specifically on the facet SFWB. We describe expected relationships among SFWB, personality, and income. We study core self-evaluations (CSE) as our personality variable because it reflects an overarching disposition reflecting many personality dimensions relevant to SWB (Judge, 2009), such as neuroticism and self-esteem (Diener et al., 1999). We go on to describe our expectations regarding the moderating effects of gender, and test our hypotheses using a nationally representative sample of twins from the Survey of Midlife Development in the United States (MIDUS). We then present our results and discuss the policy implications of our findings, including financial education and methods for building self-regulatory capacity. We note the importance of focusing on non-economic methods of bolstering well-being and encourage research into the malleable antecedents of SFWB.

### Subjective Well-being

Subjective well-being is an overall appraisal of one’s life, as well as the experience of positive emotions and the absence of negative emotions (Diener, 2000), with some researchers focusing more on the former (Ryan and Deci, 2001) and others more on the latter (Kahneman, 1998, 2000). With a focus on decision making, economists conceptualize SWB as a measure of utility (Kahneman, 2000; Kahneman and Krueger, 2006), while the psychologists’ mindset more directly situates it as aggregated moment-to-moment experiences (Kahneman, 1999) and/or as an attitude—life satisfaction—with hedonic and eudaimonic components (Deci and Ryan, 2000).

Experienced utility is akin to moment-to-moment affect and affect intensity (Kahneman and Thaler, 2006), such that expectations of changes in utility are proposed as being possible guides to decision-making (Kahneman and Snell, 1990). In psychological terms, attitudes have affective and cognitive components regarding the object or subject being evaluated, and as in utility formations there is a behavioral component in relation to the choice tendencies produced by attitudes (Diener, 2000). While measuring SWB as experienced utility or more as an attitude has been the subject of debate (and with good reason; see Kahneman et al., 1993), most SWB researchers—economists and psychologists—have tended to use global measures that are meant to capture both (Kahneman, 2000).

Although various fleeting influences can impact ratings of well-being, such as affective states (Schwarz and Strack, 1999) or an individual’s focus at a given moment (Schkade and Kahneman, 1998; Kahneman et al., 2006), there are consistent predictors of SWB at the national and individual levels. For example, contextual influences like political freedoms and other government characteristics influence SWB (Diener et al., 1995; Frey and Stutzer, 2000; Helliwell, 2003). From a dispositional standpoint, SWB is associated with personality and trait-level affect (Diener and Lucas, 1999). Also at the individual level, SWB is influenced by factors such as marriage and friendship networks (Nezlek, 2000; Headey and Wooden, 2004; Winkelmann, 2005), religiosity (Soydemir et al., 2004), health (Borooah, 2006), and things as diverse as how often people move during their younger years (Oishi and Schimmack, 2010).

Another predictor of SWB—and the focus for economists—is income and wealth at the national and individual level. Given “commonsense” ideas about how income should affect SWB through increasing levels of choice (Schwartz, 2004), early research into the income-SWB relationship proved perplexing. In a classic paper, Easterlin (1974) showed that increasing national wealth had marginal effects on SWB. This partially mirrors the finding that after moderate levels of national wealth are reached the relationship diminishes substantially (Diener et al., 1995). Further, the effect of income on SWB at the individual level is small within wealthy countries (Diener, 2000), with income accounting for only 4% of the variance in SWB in the U. S. (Easterlin, 2001). Also, upward moves in income do not appear to result in lasting changes in SWB (Brickman et al., 1978; Burchardt, 2005)—a fact that people tend not to anticipate (Loewenstein and Schkade, 1999; Kahneman and Thaler, 2006).

An explanation of these findings is that: (a) after low levels of national wealth are accrued the benefits of an increase is marginal because, (b) people evaluate themselves in a relative sense to those around them, and (c) they tend to adapt (or habituate) to changes in their circumstance, altering their targets for relative comparison and their aspirations accordingly (and in a non-linear fashion; see Frey and Stutzer, 2002a; Clark et al., 2008). Regarding the first point, national wealth is associated with important contextual factors such as health care and the ability of obtain basic necessities (Diener et al., 1995), meaning that at the national level reaching a “cut-off” point is most important for well-being (Callan and Nolan, 1991), or “livability” (Veenhoven, 1995). However, increases in income beyond this point net marginal increases (although see Deaton, 2008), with no meaningful increases in well-being beyond a per capita GDP half that of the U. S. in 1995 (Helliwell, 2003). This is because after fulfilling basic needs, norms for material possessions collectively shift upward as wealth increases. Thus, increases in wealth are balanced by increases in normative levels of wealth and material possessions (Easterlin, 1995).

Regarding the latter two points (points b and c), both are a part of a comparison process that involves comparisons to other people, or referent-others, as well as to oneself in the past and a desired future (Michalos, 2008). The former of these (point b) is called the comparison income effect by economists and discussed in terms of interdependent preferences (Ferrer-i-Carbonell, 2005), while for psychologists the analogous formulation is termed a process of social comparison (Easterlin, 2003). When evaluating ourselves relative to others we take into account our social position in terms of factors such as income and material possessions (Clark and Oswald, 1996). This means that as the wealth of an entire nation grows uniformly across its citizens SWB will not change for the nation as a whole, while higher income *within* a country may provide higher levels of SWB because it is a higher level of income relative to referent-others (Luttmer, 2005). Income and what it affords are important status markers, allowing relative comparisons that can bolster SFWB because of the ability to conspicuously consume positional goods, which create status differences through their indication of ability and worth (Veblen, 1899; Berger et al., 1972; Hirsch, 1976; Frank, 1985). However, as the referent-others used for social comparisons are adjusted upward as income and the material possessions derived from it shift, this will attenuate the income-SWB relationship (Rayo and Becker, 2005)—indeed research shows that material aspirations are highest among those with high income (Ahuvia and Wong, 2002), and such aspirations have a negative impact on well-being (Kasser and Ryan, 1993).

Similarly, the latter of these two (point c) is described by economists in terms of habit formation and in psychological terms by (hedonic) adaptation (Easterlin, 2003). Effectively, when an individual experiences a change in income the new level of income (and what it affords) is contrasted against previous income and aspirations for future income, and allows consuming new goods and services (Easterlin, 2001; Tekleab et al., 2005). SWB increases because new income is higher than previous income and is closer to income aspirations (Kahneman, 1999), and new goods and services are being consumed. However, these changes can be short lived and the day-to-day hassles of ordinary life regain their potency while people adapt to their new circumstances, alter their aspirations accordingly (Frey and Stutzer, 2002b; Stutzer, 2004)—a state of affairs dubbed the hedonic treadmill (Brickman and Campbell, 1971) or preference drift (van Praag, 1971)—which serves as a foundation for the notion of a baseline or setpoint level of SWB (Frederick and Loewenstein, 1999; Waterman, 2007).

In summary, as Frey and Stutzer (2002a; p. 415) aptly note, “the upward adjustment of aspirations induces human beings to accomplish more and more. They are never satisfied… Wants are insatiable. The more one gets, the more one wants.” Although greater income and income changes are associated with an increased ability to enhance SWB through social comparison, goal attainment, and the consumption of goods, these effects are small—even when high income and material possessions are highly valued in a culture (Clark, 2003). This means that our objective circumstances can be less important in shaping our perceptions and states of being than the stable individual differences that underlie homeostatic levels of SWB (Diener and Lucas, 1999; Cummins, 2000)—although this does vary across people, it tends to hold on average (Lucas et al., 2003; Fujita and Diener, 2005).

This state of affairs begs further questions regarding the effect of personality and income on SWB in light of its very general nature as well as its stability. First, SWB is an overall measure of well-being and therefore has a variety of facets that, while they impact decision making, will be more or less removed from direct economic decisions and circumstances. In order to better understand economic decision making in relation to income and personality we assess a form of utility more directly related to economic utility in the form of SFWB, which has been shown to correlate more strongly with income than other forms of well-being (Diener and Oishi, 2000) and act as a mediator in the income-SWB relationship (George, 1992; Schyns, 2000).

We also seek to further explore the underlying factors impacting the relationship between well-being and both personality and income for men and women. Given that personality is both genetically and environmentally influenced (Tellegen et al., 1988; Kandler et al., 2010), the relative strength of the genetic and environmental effects of personality on well-being is important to consider (DeNeve and Cooper, 1998) because well-being and its components are strongly affected by genes (Tellegen et al., 1988; Arvey et al., 1989; Lykken and Tellegen, 1996). Similarly, it has long been known that income is a function of heritable traits (Taubman, 1976), yet it is often overlooked that the genetic and environmental influences on income may exert separate effects on well-being through income. To the extent that the effects of income and personality on well-being are genetic this would help explain well-being’s stability over time, as well as shed light on the extent to which changes in environment can influence well-being (Diener et al., 2006). Finally, given the differentiation of gender roles in society it is possible that men will show a stronger effect of income on well-being than women, and the extent that such a difference is attributable to genetic and/or environmental factors will shed light on the genetic basis of sex differences in the correlates of well-being.

Specifically, when theorizing about the well-being people derive from their economic condition, the possibility of moderating variables is important to consider. As Diener and Biswas-Diener (2002; p. 131) note, “psychological factors such as needs, desires, and role might play a critical role in the relation between money and SWB,” and gender is a prime candidate in such an equation because males and females are differentiated in the societal roles they are expected to fulfill (Bem, 1974; Eagly et al., 2000)—although the strength of these differences is weakening over time, the qualities that define gender roles are relatively stable (Auster and Ohm, 2000).

Typical gender roles have females oriented toward behaviors and goals related to nurturing, with more of a focus on domestic rather than work-related activities. In contrast, traditionally, males are likely to be more focused on obtaining economic resources and using such resources to provide for a family as well as to indicate high status (Bem, 1974). Good performance in each of these domains is appreciated or punished by society to the extent that it is concordant with the gender role to which an individual is expected for conform—men acting like men are looked favorably upon while women acting like men may be punished (Eagley et al., 2003). Because the male gender role emphasizes economic success as well as the ability to procure the resources that it purchases, income should be more likely to influence his well-being than for a woman. Limited support for such an idea exists in the form of differential relationships among the income-SWB relationship for men versus women (Diener and Biswas-Diener, 2002) and the negative effects of a man not feeling like the economic “breadwinner” in a household (Crowley, 1998). However, no research to date has investigated the extent to which an income-SFWB effect is genetically and environmentally influenced across the genders.

From a socialization perspective, children learn their appropriate gender role through parenting, social exchanges, and interactions with surrounding institutions and their hierarchies. Alternatively, more biologically and evolutionary oriented approaches emphasize that males and females are hard-wired for different cognitions, affective experiences, and therefore behaviors in relation to their environments because of sex differences in hormone levels and neurological development (Bussey and Bandura, 1999; Nicholson and de Waal-Andrews, 2005). To the extent that biological factors influence gender roles it is reasonable to assume a larger genetic effect of income on SFWB for men than women. However, there are mixed findings regarding the extent to which sex differences are environmentally induced, and both genetic and environmental influences are relevant to consider (Eagly and Wood, 1999). Therefore, we do not explicitly hypothesize differential genetic and environment effects in the income-SFWB relationship across genders. However, consistent with gender role theory we do hypothesize a stronger relationship for men than women between the common genetic and environmental components of income and SFWB.

Generally, understanding the genetic and environmental influences on well-being is important (Inglehart and Klingemann, 2000). Financial remuneration is a primary method of compensation in organizations and policy makers keep a constant eye on increasing income and wealth by, for example, changing tax regimes and labor laws. If the genetic factors that underlie both personality and income account for any effects that personality and income have on well-being, this casts doubt on the long-term effectiveness of public policy and organizational practices meant to alter well-being via simplistic changes in income. If genetic factors play a major role in the relationship among income and well-being, this indicates that policy and interventions should, perhaps, take individual differences seriously. We now discuss SFWB.

### Subjective Financial Well-being and Study Hypotheses

As with other well-being concepts, for economists, SFWB may be thought of as a form of utility (or ‘welfare’; see Chan et al., 2002; Takeda, 2010), whereas for psychologists it may be conceptualized as an attitude or a facet of SWB (Ng and Diener, 2014). Relying on the model of well-being summarized in Clark et al. (2008), we can situate SFWB as being held invariant in wealthy nations as they growth wealthier over time (for similar thought see Lamale, 1958). Instead, SFWB may be more a function of one’s relative financial standing within a society (Vera-Toscano et al., 2006). Although comparisons to referent-others may be made based on objective factors, standards for relative comparison are idiosyncratic (van Raaij, 1981; Ackerman and Paolucci, 1983; van Praag and Frijters, 1999). These comparisons and the standards on which they are based serve to inform an overall level of satisfaction with one’s financial situation, expectation of one’s future financial condition, as well as perceptions of financial strains and needs in relation to earnings (Clark, 2003).

Dispositions and other individual difference characteristics serve to shape perceptions of well-being as it relates to economic standing, guided by idiosyncratic interpretations of economic needs, aspirations, and expectations (Crawford et al., 2002; Norvilitis et al., 2006). Enduring cognitive and affective tendencies shape perceptions of (a) the resources required to cope with circumstance (Sumarwan and Hira, 1993), (b) the extent to which people compare their own resources against those of others in a downward or upward fashion (Ryff et al., 1999), (c) how comparisons and predictions are made that influence the creation of desired and expected states (Loewenstein and Frederick, 1997; Schkade and Kahneman, 1998), as well as (d) other processes that concurrently take into account perceptions of one’s self-worth and capabilities to achieve various ends (Peterson, 1999; Joo and Grable, 2004). In effect this means that the factors objectively shaping one’s economic well-being should act as input into SFWB, but they tell only a part of the story. Factors underlying well-being’s consistency are important to consider (Lyubomirsky and Tucker, 1998; Lyubomirsky and Ross, 1999; Ng and Diener, 2014).

Personality and trait-level affect are implicated in well-being due to their influence on the interpretation of environmental stimuli, information processing, and decision making (Kim-Prieto et al., 2005)—there are individual differences in perceptions of objectively equivalent circumstances (DeNeve, 1999; Diener and Lucas, 1999). High levels of self-esteem and self-efficacy can bolster responses to threatening situations and failures by keeping people positive and motivated to meet challenges (Kammeyer-Mueller et al., 2009). Similarly, an internal locus of control imbues people with a sense that they have the agency required to shape both their environment and future (Furnham, 1986), and that they are not subject to uncontrollable environmental forces (Judge et al., 2004). Low levels of neuroticism reduce responses to stressful circumstances and life events and increase optimistic judgments of the future (Larsen and Ketelaar, 1991; Zelenski and Larsen, 2002), and are associated with motivation to approach positive outcomes, rather than the less effective tactic of avoiding failure (Johnson et al., 2008).

All of these traits have been shown to have a relationship with well-being (Diener and Diener, 1995; Carver and Scheier, 1999; Peterson, 1999; Tsaousis et al., 2007), including that related to work-family satisfaction (Boyer and Mosley, 2007), employment (Judge and Larsen, 2001), and SFWB beyond that of income (Judge et al., 2009; Ng and Diener, 2014). Further, these individual difference variables—neuroticism, self-esteem, generalized self-efficacy, and locus of control—have been shown to exhibit substantial heritability and consistency over time (Trzesniewski et al., 2003; Neiss et al., 2006). Also, the interrelationships among them can be describes as being a function of a higher-order factor (Judge et al., 2002). Based on their consistency, heritability, and strong interrelationships, a personality variable labeled CSE has been posited as being a primary personality variable that that reflects one’s evaluation of self-worth, capabilities, and competence (Judge, 2009), and it is implicated in well-being (Judge et al., 2005).

Core self-evaluations are distinct from well-being, utility, or happiness in that these all relate to satisfaction with life or affective experience directly (Easterlin, 2003). Alternatively, while CSE act as antecedents to well-being, they are not a part of the construct itself (Judge et al., 1998; see similar thought in Diener and Diener, 1995; Ryan and Deci, 2001). Given the relationship among the components of CSE and well-being, we expect a positive relationship between CSE and SFWB. Also, given the heritability of both well-being and personality, we expect genetic factors to play a role in the personality-SFWB relationship.

Hypothesis 1: The positive association between CSE and SFWB is due to both common genetic and environmental influences.

Apart from personality, the role that income plays in SFWB is important to consider given the clear importance of income in understanding one’s economic condition (Buchler et al., 2009). Although processes of adaptation and changing referent-others should reduce the impact of income on SFWB, income still plays a role in economic well-being (Diener and Biswas-Diener, 2002; Clark et al., 2008). Although the discussion of the income-SWB relationship above would seem to indicate that income might not have a strong effect on SFWB, the relative comparisons made when evaluating SFWB should be based more on economic factors than when evaluating overall well-being. Overall evaluations of SWB take into account the many facets that make up well-being, such as family and other social relationships, job satisfaction, physical health, and the like (Diener and Seligman, 2004). In contrast, SFWB is focused on the subjective evaluation of one’s economic situation, and therefore relative comparisons should be based more on income-relevant factors (Vera-Toscano et al., 2006). Therefore, we expect that income will have a positive effect on SFWB.

However, in this relationship, it is important to understand the extent to which both income and SFWB are a function of environmental and genetic influences that will in turn drive their association. Income is heritable (Taubman, 1976; Bowles and Gintis, 2002), as are most of its antecedents, such as risk preferences (Zyphur et al., 2009), entrepreneurialism (Nicolaou et al., 2008; Zhang et al., 2009), financial management behaviors (Barnea et al., 2010), and the factors that shape job performance, such as cognitive ability (McGue and Bouchard, 1998). Further, more basic and highly heritable traits such as height and attractiveness are related to income (Hosoda et al., 2003; Judge and Cable, 2004). Given these relationships, it is possible that the same genetic and environmental factors that influence income could also drive well-being. In other words, it is possible that the influence of income on SFWB may be a function of both common (i.e., the same) genetic and environmental factors.

Hypothesis 2: The association between income and SFWB is due to both common genetic and environmental influences.

Consistent with our earlier theorizing, we propose that the strength of the genetic and environmental associations among our variables may be moderated by gender.

Hypothesis 3: As compared with women, men exhibit stronger relationships between the genetic components of income and SFWB, and between the environmental components of these two variables.

## Materials and Methods

### Sample

The twins sample was drawn from MIDUS, which is a nationally representative sample of the U. S. surveyed in accordance with research ethics regulations (see Kessler et al., 2004). The national twins sample in MIDUS includes 998 twin pairs (25–74 years old). We used the 712 same-sex twin pairs with data along CSE, income, SFWB, and control variables. This sample contains 170 monozygotic (MZ or identical) male twin pairs, 194 MZ female pairs, 135 dizygotic (DZ or fraternal) male pairs, and 213 DZ female pairs. Their average age was 44.66 (ranging from 25 to 74). The data used for our study were collected from 1996 to 1997.

### Measures

#### Subjective Financial Well-being

Subjective financial well-being was measured by five items asking participants about their financial situation. Examples include, “How would you rate your financial situation these days?” (0 = the worst possible financial situation to 10 = the best possible financial situation), “Looking ahead 10 years into the future, what do you expect your financial situation will be like at that time” (0 = the worst possible financial situation to 10 = the best possible financial situation), “In general, would you say you (and your family living with you) have more money than you need, just enough for your needs, or not enough to meet your needs” (1 = more money than you need to 3 = not enough money, reverse coded). Cronbach’s alpha was 0.74.

The measure’s construct validity was supported by exploratory and confirmatory factor analyses (CFA). We randomly split the sample in half and exploratory analyses on the first half showed that one factor accounted for 55.98% of the total variance, with an Eigen value = 2.80 and no other factors with an Eigen value > 1. We conducted CFA on the second half and found that the one-factor model fit the data acceptably well [χ^2^ = 51.84, df = 5, *p* < 0.001, Tucker-Lewis Index (TLI) = 0.91, Comparative Fit Index (CFI) = 0.95, Root Mean Square Error of Approximation (RMSEA) = 0.12, Standardized Root Mean Square Residual (SRMR) = 0.04].

#### Core Self-evaluations

Core Self-Evaluations was measured using a 15-item scale developed by Judge et al. (2009) based on the existing items in the MIDUS database. This is because the MIDUS data collection was initiated (around 1996 to 1997) before Judge et al. (2003) CSE scale was developed. This 15-item scale has been shown to have high reliability and convergent validity with the original CSE scale (see Judge et al., 2009 for its construct validity information). Example items include, “I often feel worthless,” “What happens in my life is often beyond my control,” “I often feel helpless in dealing with the problems of life” (reversed). Because the 15 items are on different scales, we standardized the responses on each item and then averaged the standardized scores to obtain a measure of CSE. The internal consistency coefficient of this measure was 0.86.

#### Income

Participants’ income was measured by an item asking participants to indicate their own personal pre-tax earnings (only wages and stipends from employment) in the past 12 months. Responses were from 36 pre-defined categories, ranging from 0 dollar to 1,000,000 dollars or more. We used the mean dollar value for a participant’s chosen range as the measure of income for the person. To reduce skewness in the measure, we took a natural logarithm transformation of the dollar amounts and used the log-transformed measure in our data analyses.

#### Gender

Gender was measured with a self-report item coded as one (female) or zero (male).

#### Control Variables

Research shows that SWB is influenced by age, education, number of children, and marital status (Diener et al., 1999), which we conrolled for in our analyses. These variables were self-reported as integers. Level of education ranged from 1 (no school/some grade school) to 12 (doctoral degree). Marital status was coded as one (married) or zero (not married).

## Results

### Discriminant Validity

A series of CFAs were done to test the discriminant validity of CSE and SFWB measures. To obtain an optimal ratio of sample size over the number of CFA parameters, we randomly generated three item parcels (five items for each parcel) for the 15-item measure of CSE. CFA results show that a two-factor model (with three parcels measuring CSE and five items measuring SFWB) fit the data adequately (χ^2^ = 100.15, df = 19, *p* < 0.001; TLI = 0.948, CFI = 0.965, RMSEA = 0.081, SRMR = 0.046), and a two-factor model fit the data better than a one-factor model (Δχ^2^ = 727.40, Δdf = 1, *p* < 0.001; for the one-factor model, χ^2^ = 827.55, df = 20, *p* < 0.001; TLI = 0.510, CFI = 0.650, RMSEA = 0.249, SRMR = 0.164). Thus, we concluded that CSE and SFWB were empirically distinct and could be treated as separate constructs in analyses (supported by the magnitude of their correlation, 0.35, as noted in **Table 1**).

**Table 1:** Descriptive statistics and zero-order correlations for study variables for males and females.

Variables	*M*_male_	*SD*_male_	1	2	3	4	5	6	7	*M*_female_	*SD*_female_
(1) Age	44.55	11.92	–	-0.15^∗∗^	-0.03	0.36^∗∗^	-0.09^∗∗^	-0.27^∗∗^	0.13^∗∗^	44.73	12.33
(2) Level of education	6.81	2.48	-0.07	–	0.01	-0.21^∗∗^	0.27^∗∗^	0.21^∗∗^	0.17^∗∗^	6.33	2.30
(3) Marital status^1^	1.23	0.42	-0.20^∗∗^	0.00	–	-0.21^∗∗^	-0.07	0.11^∗∗^	-0.11^∗∗^	1.31	0.46
(4) Number of children	1.91	1.42	0.39^∗∗^	-0.15^∗∗^	–	–	-0.05	-0.23^∗∗^	-0.07^∗^	1.96	1.42
(5) Core self-evaluations (CSE)	0.04	0.57	0.04	0.18^∗∗^	0.03	0.02	–	0.13^∗∗^	0.33^∗∗^	-0.04	0.62
(6) Income (natural-log transformed)	9.53	2.91	-0.36^∗∗^	0.25^∗∗^	-0.06	-0.05	0.14^∗∗^	–	0.06	7.68	3.83
(7) Subjective financial well-being (SFWB)	0.02	0.68	0.19^∗∗^	0.12^∗∗^	–	0.06	0.39^∗∗^	0.15^∗∗^	–	-0.02	0.79

*N = 528–591 individuals. ^1^1=currently married, 0=otherwise. ^∗^*p* < 0.05, ^∗∗^*p* < 0.01 (two-tailed). Values in the upper diagonal are correlation coefficients for females and values in the lower diagonal are correlation coefficients for males.*

### Twin Analyses

**Table 1** presents means, SDs, and correlations of the study variables. Within-pair correlations (i.e., the correlations between the first and second twin in a pair) are displayed in **Table 2**—upper-diagonal values are for MZ pairs and lower-diagonal values are for DZ pairs.

**Table 2:** Within-twin-pair correlation for the variables for males and females.

Variables	Male		Female	
	MZ	DZ	MZ	DZ
(1) CSE	0.37^∗∗^	0.28^∗∗^	0.58^∗∗^	0.28^∗∗^
(2) Income	0.51^∗∗^	0.31^∗∗^	0.31^∗∗^	0.24^∗∗^
(3) Subjective financial well-being	0.32^∗∗^	0.21^∗∗^	0.30^∗∗^	0.23^∗∗^

*N = 364 MZ twin pairs and 348 DZ twin pairs. ^∗∗^*p* < 0.01 (two-tailed).*

Behavioral genetic analyses were done as described by Plomin et al. (2013). We used a two-stage multi-group structural equation modelling (SEM) strategy: we first conducted a series of univariate analyses on the three variables of interest (SFWB, CSE, and income) and then we performed multivariate analyses on the three variables simultaneously (as in Neale and Cardon, 1992). The SEM program Mplus version 7 was used, with SFWB and CSE modeled as latent variables for all analyses.

Univariate analyses were conducted to estimate the percentage of variance in SFWB, CSE, and income that can be attributed to three factors: additive genetic factor (A), shared environmental factor (C, representing shared rearing environment for all siblings and unique environmental factor (E, e.g., developmental and other environments and experiences that are unique to an individual). Specifically, any variable P can be modeled as

$$\text{P}\;\text{=}\;{\text{a}}^{\text{*}}\text{A}\;\text{+}\;{\text{c}}^{\text{*}}\text{C}\;\text{+}\;{\text{e}}^{\text{*}}\text{E}$$

where A, C, and E refer to the latent genetic, shared environmental, and unique environmental factors (including potential measurement errors), respectively, with means of zero and variances of 1. In turn, a, c and e refer to the corresponding path coefficients.

The relative influence of the genetic factor can be obtained by dividing the variance accounted for by A by the total variance in variable P: a^2^/(a^2^ + c^2^ + e^2^), which is defined as the heritability of the variable. Similarly, the relative influence of the shared environmental factor is c^2^/(a^2^ +c^2^ +e^2^) and for the unique environmental factor it is e^2^/(a^2^ + c^2^ + e^2^). It is notable that because CSE and SFWB were modeled as latent variables with multiple indicators, their variance components do not contain measurement errors.

We conducted univariate analyses on the three study variables for males and females. Prior behavioral genetics research often shows a lack of influence for the shared environmental factor C (e.g., Turkheimer, 2000; Johnson and Krueger, 2006). Thus, we compared three nested models to identify the best fitting model for each of the study variables for males and females: an ACE model with all the three factors; an AE model; and an E model. Such a model-fitting approach allows “different types of models to be explicitly tested and compared” (Plomin et al., 2013, p. 384) in order to identify the most parsimonious model “with the smallest number of parameters that generates expectations that match the observed data as closely as possible” (p. 383). We did not examine an ‘ADE’ model with a dominant genetic factor D because correlations among MZ twins were not more twice that of DZ twins (see **Table 2**), which is a precondition for identifying a dominant genetic effect. Results (see **Table 3**) support genetic A and unshared environmental E influences on CSE, income, and SFWB for both males and females, but not shared environmental influences C.

**Table 3:** Univariate model fitting for CSE, income, and subjective financial well-being for males and females.

Model	Model fit indices						
	χ^2^(df)	Δχ^2^	RMSEA	SRMR	AIC	TLI	CFI
**CSE**							
Male (*h*^2^ = 40.2%)							
Model 1: A,C,E	40.40 (30)	–	0.049	0.095	2134.12	0.99	0.99
Model 2: A,E^@^	41.92 (31)	1.52	0.049	0.101	2133.64	0.99	0.99
Model 4: E	69.03 (32)	28.63^∗∗∗^	0.089	0.187	2158.76	0.96	0.96
Female: (*h*^2^ = 64.4%)							
Model 5: A,C,E	34.46 (30)	–	0.027	0.052	3265.76	1.00	1.00
Model 6: A,E ^@^	34.46 (31)	0.00	0.024	0.052	3263.76	1.00	1.00
Model 8: E	118.80^∗∗∗^ (32)	84.34^∗∗∗^	0.116	0.221	3346.12	0.94	0.94
**Income**							
Male (*h*^2^ = 55.9%)							
Model 1: A,C,E	12.90^∗^(6)	–	0.089	0.315	2582.71	0.86	0.95
Model 2: A,E^@^	12.90 (7)	0.00	0.077	0.315	2580.71	0.88	0.97
Model 4: E	64.86^∗∗∗^ (8)	51.96^∗∗∗^	0.223	0.363	2630.68	0.00	0.71
Female: (*h*^2^ = 33.1%)							
Model 5: A,C,E	4.92 (6)	–	0.000	0.102	3775.68	1.00	1.01
Model 6: A,E ^@^	6.21 (7)	1.29	0.000	0.106	3774.96	1.00	1.01
Model 8: E	29.27^∗∗∗^ (8)	24.35^∗∗∗^	0.117	0.163	3796.03	0.07	0.77
**SFWB**							
Male (*h*^2^ = 42.3%)							
Model 1: A,C,E	53.63^∗∗^ (30)	–	0.074	0.085	3441.40	0.96	0.96
Model 2: A,E^@^	53.67^∗∗^ (31)	0.04	0.071	0.085	3439.45	0.96	0.96
Model 4: E	71.67^∗∗∗^ (32)	18.04^∗∗∗^	0.092	0.141	3455.44	0.93	0.93
Female: (*h*^2^ = 27.7%)							
Model 5: A,C,E	45.07^∗^ (30)	–	0.050	0.047	5234.71	0.98	0.98
Model 6: A,E^@^	45.38^∗^ (31)	0.31	0.048	0.049	5233.02	0.98	0.98
Model 8: E	57.57^∗∗^ (32)	12.50^∗∗^	0.063	0.095	5243.21	0.97	0.97
							

*N = 364 and 348 pairs for identical and fraternal twins, respectively. A, C, and E denotes additive genetic factor, shared environmental factor and unique environmental factor, respectively. *h*, heritability estimate. RMSEA, Root mean square error of approximation; AIC, Akaike’s Information Criterion; TLI, Tucker–Lewis Index; CFI, Comparative fit index. SRMR, Standardized Root Mean Square Residual, ^@^Indicates the best fit model. ^∗^*p* < 0.05, ^∗∗^*p* < 0.01, ^∗∗∗^*p* < 0.001.*

**FIGURE 1:**
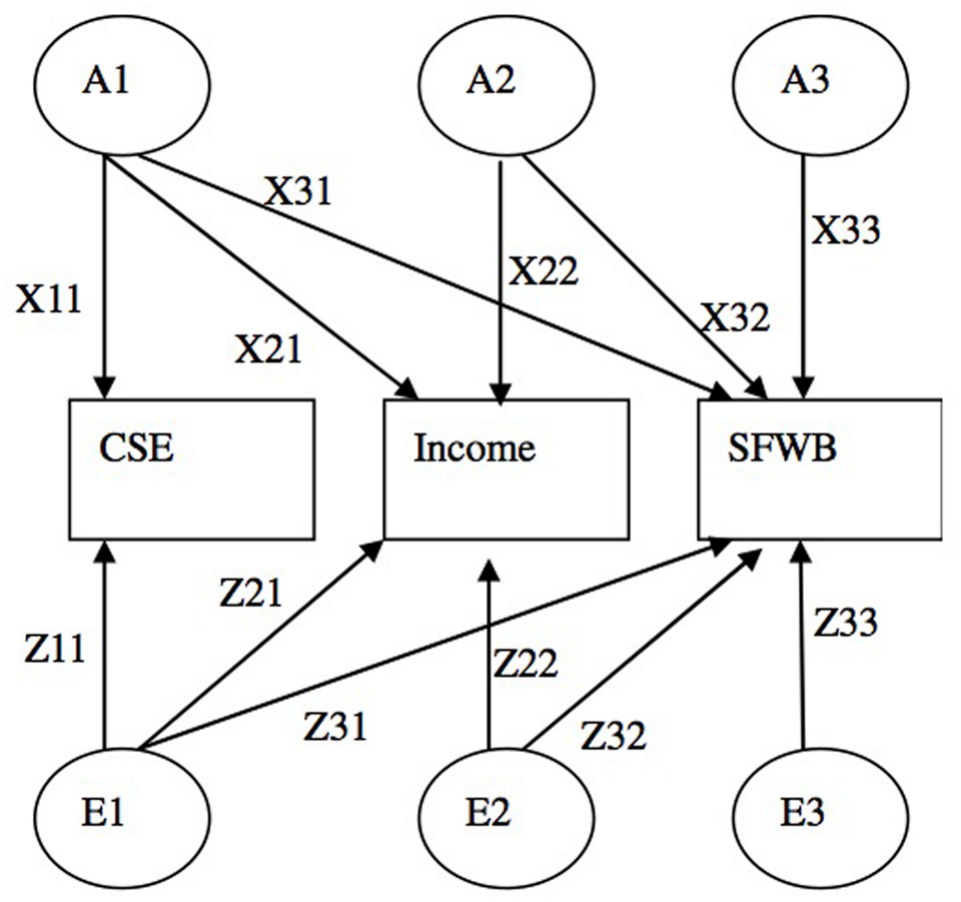
**Multivariate multi-group structural equation model for core self-evaluations (CSE), income and subjective financial well-being (SFWB).** This figure displays a partial diagram with additive genetic factors (A1, A2, and A3) and unique environmental factors (E1, E2, and E3) for one twin only. Shared environmental factors (C1–C3) are not modeled because univariate analyses show that their influences are not significant. For simplicity purposes, control variables are not shown in the figure. CSE and SFWB are measured as latent variables with multiple indicators.

We then conducted multivariate behavioral genetics analyses on the three variables using Cholesky decomposition to estimate the influences of CSE and income on SFWB (see Neale and Cardon, 1992; see **Figure 1** for a graphical depiction). This decomposition is analogous to a hierarchical regression wherein personality is first entered as a primary predictor, with income entered second and its effect understood in terms of change in *R*^2^ (although in our case we assess the effects of behavioral genetics factors associated with personality versus income). Because the results of univariate analyses suggest that only A and E factors play a role in impacting the three variables, we fixed the shared environmental factor C to zero in subsequent analyses (as recommended in Plomin et al., 2013). As shown in **Figure 1**, we label the common (overlapping) genetic factor between CSE and SFWB as A1 and the common genetic factor between income and SFWB as A2. Likewise, the common (overlapping) environmental factor between CSE and SFWB is E1 and the common environmental factor between income and SFWB is E2.

**FIGURE 2:**
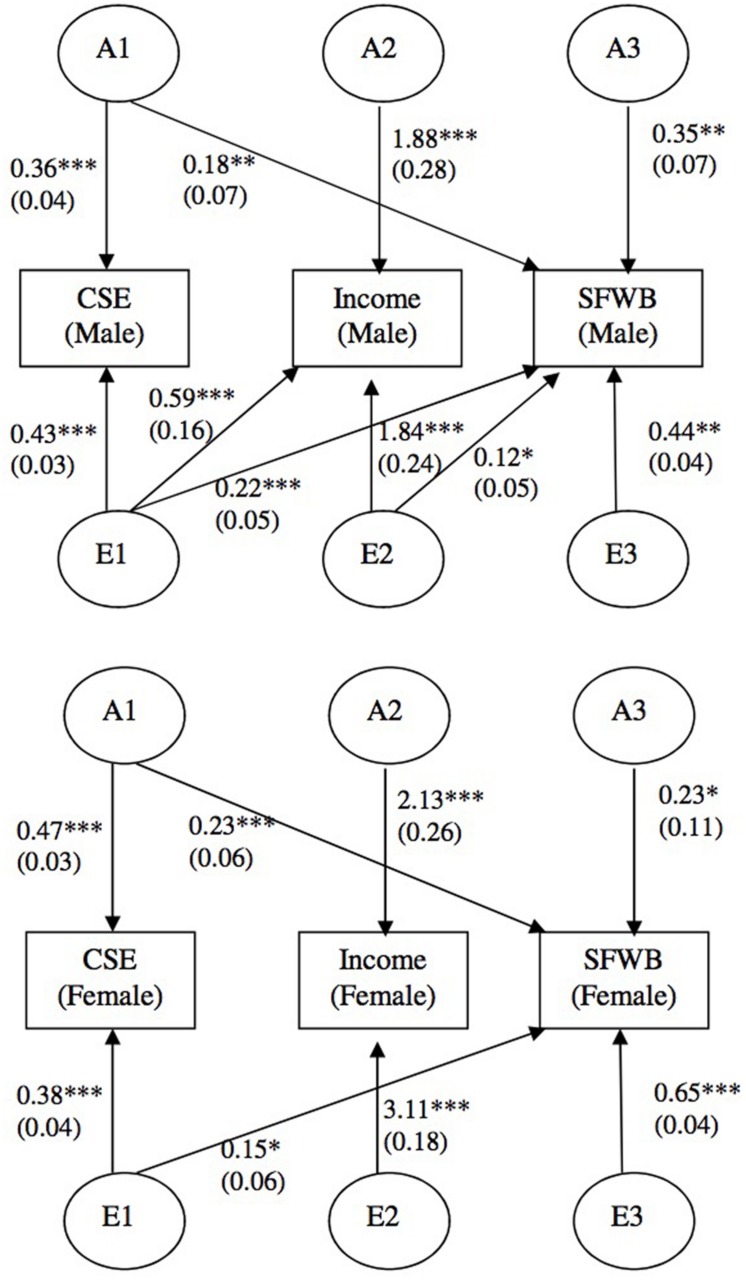
**Results of multivariate analysis for males and females.** Unstandardized path coefficients with their standard errors (in parentheses) are reported. For simplicity purposes, control variables are not shown. CSE and SFWB are measured as latent variables with multiple indicators. ^∗^*p* < 0.05, ^∗∗^*p* < 0.01, ^∗∗∗^*p* < 0.001.

Including all control variables as predictors, we first estimated a model for females and a model for males separately, before testing a combined model for both gender groups. **Table 4** shows the fit of the separate and combined models. For males, the AE model for the three study variables adequately fit the data (Model 1 in **Table 4**). Results indicate that two paths, x21 (the effect of A1 on income) and x32 (the influence of A2 on SFWB), were not significant. Accordingly, as recommended in Plomin et al. (2013), we constrained these two paths to zero (Model 2, **Table 4**), which did not cause meaningful decrements in fit compared to Model 1. For females, the model with both A and E factors for all of the three study variables also showed adequate fit (Model 3, **Table 4**) and four paths were not significant: x21 (the effect of A1 on income), x32 (the influence of A2 on SFWB), z21 (the effect of E1 on income), and z32 (the impact of E2 on SFWB). Therefore, the four paths were constrained to zero in a nested model (Model 4, **Table 4**), which also did not cause meaningful decrements in fit compared to Model 3. Consequently, the best multivariate models were obtained for both males (Model 2) and females (Model 4). We then combined the two best fitting models into a single model (Model 5). Results show that the combined model did not achieve exceptional fit, but the fit was adequate to examine the effects contained in the combined model. We tested our hypotheses based on the path coefficients in this combined model, as presented in **Figure 2**.

**Table 4:** Multivariate model fitting for CSE, income, and subjective financial well-being for males and females.

Model	Model fit indices						
	χ^2^ (df)	Δχ^2^	RMSEA	SRMR	AIC	TLI	CFI
**Males**					
Model 1: A and E factors for all the three variables	496.06^∗∗∗^ (367)	–	0.051	0.088	14448.79	0.93	0.93
^@^Model 2: Model 1 plus x21 = 0, and x32 = 0	498.43^∗∗∗^ (369)	2.37	0.051	0.088	14447.16	0.93	0.93
**Females**							
Model 3: A and E factors for all the three variables	574.26^∗∗∗^ (367)	–	0.055	0.074	21029.94	0.92	0.92
^@^Model 4: Model 3 plus x21 = 0, x32 = 0, z21 = 0, and z32 = 0	578.42^∗∗∗^ (371)	4.16	0.055	0.075	21026.10	0.92	0.92
**Combined model for both males and females**					
Model 5: combination of Model 2 and Model 4	1224.60 ^∗∗∗^ (771)	–	0.060	0.092	35559.01	0.90	0.90

*N = 364 and 348 pairs for identical and fraternal twins, respectively. A, C, and E denotes additive genetic factor, shared environmental factor and unique/non-shared environmental factor, respectively, RMSEA, Root mean square error of approximation; AIC, Akaike’s Information Criterion; TLI, Tucker–Lewis Index; CFI, Comparative fit index. SRMR, Standardized Root Mean Square Residual, ^@^ Indicates the best fit model. ^∗∗∗^*p* < 0.001.*

Hypothesis 1 predicted that there are common genetic and environmental influences between CSE and SFWB. The results in **Figure 2** show that for both males and females, the common genetic factor and the common environmental factor between CSE and SFWB have significant influence on both variables, after partialling out the influences of control variables (i.e., age, education, marital status, and number of children). For males, the common genetic factor accounted for 7.9% [=0.182/(0.352 + 0.182 + 0.222 + 0.122 + 0.442)] of the total variance in SFWB, and the common unique environmental factor explained 11.8% [(=0.222/(0.352 + 0.182 + 0.222 + 0.122 + 0.442)] of the variance in SFWB. These are akin to correlations of 0.28 and 0.34 among the genetic and environmental factors, respectively, for CSE and SFWB. For females, the common genetic factor accounted for 9.6% (=0.232/(0.232 + 0.232 + 0.152 + 0.652)] of the total variance in SFWB, and the common unique environmental factor explained 4.1% [=0.152/(0.232 + 0.232 + 0.152 + 0.652)] of the variance in SFWB. These are akin to correlations of 0.31 and 0.20 among the genetic and environmental factors, respectively, for CSE and SFWB. Thus Hypothesis 1 was supported.

Hypothesis 2 proposes that common genetic and environmental influences exist between income and SFWB. Results of the combined model reveal that for both males and females, there is no common genetic factor that simultaneously has impact on both income and SFWB, after partialling out the control variables. The overlapping unique environmental factor only exists for males, which explains 7.6% [=(0.122 + (0.22^∗^0.59)2)/(0.352 + 0.182 + 0.222 + 0.122 + 0.442)] of the total variance in SFWB, depicting a purely environment-driven correlation between income and SFWB of 0.28. It is notable that because of our Cholesky decomposition we must include in the numerator a term representing the variance in income accounted for by CSE that is shared with SFWB (i.e., z31^∗^z21). For the interested reader, the relationship between income and SFWB controlling for personality is computed by removing this term, which is 3.5% [=0.122/(0.352 + 0.182 + 0.222 + 0.122 + 0.442)], which is a partial correlation of 0.19. For females, there is no overlapping environmental factor that influences income and SFWB. Thus, Hypothesis 2 was partially supported for males only regarding the common environmental influences (see **Figure 2** for path coefficients and statistical significance).

Hypothesis 3 predicted that the overlapping genetic and environmental factors between income and SFWB show a different pattern of relationships for men versus women. Results concerning this hypothesis can be examined from both a common genetic (A) and environment (E) standpoint. For both men and women, the final model showed no overlapping genetic factor between income and SFWB; but there is an overlapping unique environmental factor between income and SFWB for men but not women (path z23 = 0.12, *p* < 0.05). This latter finding of a difference in the pattern of relationships supports part of Hypothesis 3, because the overlapping unique environmental factor is significant and was retained in the final model for men, but not for women (both with and without the effect of personality partialled out of the relationship; **Figure 2** shows path coefficients and statistical significance).

## Discussion

Examining a type of well-being that should be closely related to economic decision making, SFWB, we confirmed that personality and income are important for understanding how people feel about their economic circumstances. However, while personality is related to SFWB for both men and women, only for men is income related to SFWB—showing a different pattern of relationships across the sexes. Also, while both genetic and environmental components of personality are related to SFWB, only environmental factors explain the income-SFWB relationship. In both cases of environmental influence, it is the unique environmental factors that drive the relationship. Unique or unshared environments are those that are not mutually experienced by siblings—for example, aspects of parenting and home life are attributable to shared environments, while unique peer groups and experiences at school, work, and elsewhere make up unshared environments. Below, we discuss our results, their practical importance, and our study’s limitations.

### Personality and SFWB

Our findings show that both genes and environment affect the relationship between CSE and SFWB. As previous research indicates, the ways we feel about ourselves in terms of our self-worth, our ability to reach goals and control our outcomes, as well as our experience of negative emotions all have implications for our well-being (Diener and Lucas, 1999), and we confirm this with SFWB. The genetic component of this relationship indicates that nature, so to speak, predisposes us in this respect: genetic factors co-influence personality and a sense of economic well-being. We are born with neuro-chemistries that simultaneously influence our personalities and our sense of well-being.

On the other hand, a part of this relationship is environmentally influenced. Each of us has a unique environment that shapes our personality and makes us different from even genetically identical siblings (Neiss et al., 2006). The environmental component underlying the CSE-SFWB relationship shows that the experiences that form our personality also influence our SFWB. The lack of a shared-environment effect indicates that upbringing and factors related to a shared home life are not necessary for explaining CSE, SFWB, and their relationship. In other words, we find no evidence of shared upbringing such as parenting effects on the relationship between personality and SFWB. Given the age of our sample, this is not necessarily a surprise. Previous research shows that shared environmental effects often become negligible as people age (Rice et al., 2002).

### Income and SFWB

The relationship between income and SFWB is more complex, and more interesting. While most studies show a cross-sectional relationship between income and well-being (Clark et al., 2008), most statistically control for gender rather than look for differences across the sexes in this relationship, and none have decomposed the genetic and environmental parts of this relationship for each sex. On this point, it appears that in terms of SFWB, money talks, but only for men. Given that men tend to place a higher value on their income-relevant accomplishments than women (Crowley, 1998), this is predictable. However, what is less predictable is that in our sample this appears to not be a function of the human genome. Our results indicate that the environmental forces that make men earn more or less money are what influence SFWB.

This finding stands in contrast to the tenets of evolutionary psychology as it relates to the genetic basis of gender roles and mating strategies across the sexes (Buss, 1994). Such approaches tend to describe the man-as-hunter, an entity who is able to reproduce and support offspring to the extent that he is able to procure resources. In theory, this naturally selects for a genetic make-up that makes man value income and the procurement of other resources—because of man-as-hunter’s genes, he has doffed his pelts, donned a suit, and now searches for lucrative stock trades because they increase his experienced utility. Juxtaposing this, our results show that the environment drives the income-SFWB relationship, meaning that an income-SFWB relationship is not being kept in man’s genes, so to speak. This finding gives credence to the argument that culture shapes our values and gender roles, and that men are not genetically predisposed toward a relationship between their income and well-being. This has relevance for multiple literatures.

First, as we described above, the linkage between income and SWB has received tremendous attention, with a modest relationship being observed in wealthy nations (Diener et al., 1995). Our findings confirm that the relationship between income and SFWB seems larger than typically found for SWB (0.28 in our study versus an average around 0.20 for SWB). However, this relationship is observed only in the males of our sample. Also, this relationship is entirely environmentally influenced and, therefore, it may be at the whim of cultural values. We do not mean to say that the high importance placed on money is not deep-seated or is capricious in most wealthy nations, but our findings do point to its malleability owing to it being environmentally influenced.

Second, our findings are a commentary on literature showing the stability of income across generations (Solon, 1992), which occurs due to monetary inheritances and shared environmental influences among siblings, as well as genetically transmitted factors that help to affect human capital, such as personality, physical features, and to some extent intelligence (Bowles et al., 2001). Given the genetic aspect of these findings, one might conjecture that the relationship between income and well-being is genetically influenced. Using logic typical of evolutionary psychology, this could be due to an evolved resource-reward “module” for men that drives higher levels of income through its effects on well-being. Our results indicate that this is not the case, and that it is neither genes nor the family environment that give men a sense of economic well-being as a function of income. Instead, it is unique experiences that cause them to link their income with understandings of their economic well-being. Generalizing our findings, this indicates that the persistence of income across generations is not due to a genetic or shared family environment link determining the relationship between income and SFWB.

### Practical Applications

Our findings point toward the importance of public policy and organizational practices in altering well-being, and SFWB specifically. Low levels of well-being are associated with a host of negative outcomes for people and society, with health and suicide being but two of them (Wheaton, 1994; Koivumaa-Honkanen et al., 2001; Helliwell, 2006). One school of thought on this state of affairs is that both policy makers and organizations have a responsibility to maximize the public’s well-being (Kahneman, 2000), with some offering a perspective of “libertarian paternalism” wherein preferences are matched in a way that is beneficial to society (Sunstein and Thaler, 2003)—although see Sugden (2004).

Literature addressing interventions to increase well-being has come from multiple angles. One example is addressing the measurement issues involved in moving from micro to more macro levels of analysis to inform decision making (see Diener, 2000; Diener and Seligman, 2004; Kahneman et al., 2004b; Kahneman and Sugden, 2005). Complementary literature by economists has identified the best targets for intervention at the national level, such as measures to increase social capital and trust, good governance, and participation in community organizations (Helliwell, 2003, 2006) as well as balancing the effects of inflation versus unemployment on well-being (Di Tella et al., 2001) and minimizing upward social comparisons (Frank, 1997). Alternatively, with a more micro approach, psychologists point to increasing feelings of gratitude, self-affirmations, and social relationships (Buss, 2000; Seligman et al., 2005), with a focus on positive emotions because these help built psychological resources that assist in daily life and can have lasting effects on well-being (Fredrickson et al., 2008).

Whatever the focus of interventions—at the national or individual level—policy makers should target not only what influences well-being most, but also what can be changed through policy. Given our findings, the most important thing to consider is that while personality and well-being are quite stable, as we show CSE and SFWB have strong environmental determinants (similar to levels observed elsewhere; see Lykken and Tellegen, 1996). The heritability of CSE is 40.2% for men and 64.4% for women while the heritability for SFWB is 42.3% for men and 27.7% for women. Because the genetic and environmental influences sum to 100%, this means that substantial portions of CSE and SFWB are influenced by the environment. Importantly, these environmental forces tend to be exerted during developmental periods (Kandler et al., 2010) when personality is least stable (Ferguson, 2010). By focusing on improving the well-being of children and teenagers it is possible to increase well-being across the life-span, perhaps with interventions focus on the factors discussed here (e.g., building social relationships and trust, organizing communities, developing positive self-regard and gratitude).

However, in recommending an approach targeting younger members of society, we do not deny the importance of attending to the well-being of all members of society using similar techniques. Given the agreement among economists and psychologists that changes in income are not likely to net large increases well-being (Diener and Seligman, 2004; Clark et al., 2008), policy makers should focus on factors that do affect well-being in adulthood. For example, separation and divorce equate to low levels of well-being (Helliwell, 2006), as does unemployment (Lucas et al., 2004; Clark et al., 2008).

More specific to SFWB, this form of well-being is directly linked to how individuals feel about their economic circumstances and outcomes. While our data are not longitudinal in nature, we assume that just as with SWB, a change in income is likely to have transient effects on SFWB. To increase SFWB will likely require increasing the knowledge and skills that people have to manage their finances (Porter and Garman, 1993). Such education should allow people a greater sense of control over their economic outcomes, important because low levels of SFWB are linked to undesirable outcomes such as depression (Zimmerman and Katon, 2005).

An additional point worth mentioning is that wide-spread education about the relationship between income and well-being at the national level has potential to increase well-being. In the U. S. and elsewhere, high levels of materialism go hand-in-hand with beliefs in a positive relationship between well-being and money (through the goods, services, and status it affords; Sirgy, 1998). Educating people on the empirically verified predictors of well-being could change these beliefs. For example, instead of striving to achieve extrinsic, materially oriented goals, espousing the positive and enduring effects of achieving intrinsic goals could be espoused (Kasser and Ryan, 2001). People could also be informed that social interactions with friends, relative, and partners (in that order) are found to be most pleasurable, while interaction with one’s boss, clients or customers, and co-workers (in that order) are associated with the greatest amount of displeasure (Kahneman et al., 2004a). This could help people adjust their values, thereby changing their behavior and, ultimately, their well-being.

### Limitations and Future Directions

Limitations of our research include the cross-sectional nature of our data. Prior research shows mixed results in the causal relationship between factors inherent in CSE, specifically self-esteem, and well-being (Baumeister et al., 2003). Although some research suggests a causal CSE-SFWB relationship (see Judge et al., 2009), the reverse cannot be ruled out. Further, income and well-being can have a bi-directional relationship (Clark et al., 2008). This makes it difficult to infer directional effects among the genetic and environmental components of our variables. However, in our study we are not making causal claims as much as we are disentangling the common genetic and environmental components of income, CSE, and SFWB—to do this does not require longitudinal data. In either case, and although this is a common call in research on well-being (Frey and Stutzer, 2002a), we echo the need for panel data to assess the causal relationships among income, personality, and well-being. In the context of common genetic and environmental components this is especially important because of the interesting effects it could net. For example, it would be possible to show a SWB → income effect for the common genetic component, while there could be an income → SWB effect for the common environmental component.

Also, while research shows small effects of income on well-being, measures of wealth appear to have stronger effects (Headey et al., 2008). Given the broad nature of SFWB and our measure of it, it is possible that overall wealth—including savings and other assets—could explain SFWB better than income alone. Future research into this relationship is warranted.

Considering the measurement of SFWB and well-being generally, there is no shortage of opinions on the best way it should be conceptualized and measured (see Lucas et al., 1996; Kahneman, 1999, 2000; Ryan and Deci, 2001; van Praag et al., 2003; Kahneman et al., 2004a; Kahneman and Krueger, 2006). In the context of examining the genetic and environmental influences on well-being, it could well be the case that broad global measures (like that used here for SFWB) exhibit lower levels of heritability than those that are moment-to-moment. The aggregate of moment-to-moment experience is likely to be a stronger corollary of neurological functioning than more global well-being measures (which are more like attitudes than experience), and such functioning is highly heritable (Piccini et al., 1999). With this in mind, our results should not necessarily be used to make inferences about the genetic and environmental effects of income or personality on momentary measures of well-being.

Next, it is notable that determining the “actual” environmental and genetic components of a given phenotype is impossible because the magnitude of these components are dependent upon the environment in which an organism develops and lives (Harris et al., 1992; Zhang et al., 2009). In environments where behavioral and psychological expressions of a genotype are unconstrained, genetic effects tend to be weaker than when they are constrained (Gottesman, 1991; Rende and Plomin, 1992). For example, entrepreneurship tends to be more heritable for women than for men (Zhang et al., 2009). In theory this is because women face greater difficulties obtaining venture capital and other forms of support, meaning that only females with a strong genetic predisposition toward entrepreneurship engage in the behavior. Were the environment to be more open in this regard, heritability levels might subside. Drawing analogy to our sample, the environment is hardly constraining in the U. S. in terms of both income and well-being. Were our sample taken from an economically impoverished country we would expect to observe the typically larger effect between income and well-being (Clark et al., 2008), and the constraints imposed in such an environment could increase the genetic component of this effect.

## Conclusion

What is commonly believed to result in happiness, such as money and material possessions, does not appear to have this effect in a lasting manner. This is sensible given that our genetic makeup appears to at least partially affect baseline levels of well-being, as well as its relationship with personality. While we might assume that the portion of well-being and its relationship with personality that are environmentally influenced could be the target of intervention, even this component of these variables tends to stabilize after adolescence. This state of affairs indicates that public policy that would alter well-being should be targeted at people during their formative years, to influence well-being and personality when it is easiest to do so. Also, based on the lack of a genetic relationship between income and SFWB (and the overall lack of a relationship) for women, it can be concluded that even men are not genetically predestined to appreciate income for its own sake (even as it relates to SFWB). Therefore, especially at younger ages, we recommend educating the public that the quest for more money is likely to lead to only one thing with some degree of certainty: more money.

## Conflict of Interest Statement

The authors declare that the research was conducted in the absence of any commercial or financial relationships that could be construed as a potential conflict of interest.
